# Trend of Survival of a Cohort of Chinese Patients With Systemic Lupus Erythematosus Over 25 Years

**DOI:** 10.3389/fmed.2020.00552

**Published:** 2020-09-11

**Authors:** Chi Chiu Mok, Ling Yin Ho, Kar Li Chan, Sau Mei Tse, Chi Hung To

**Affiliations:** Department of Medicine, Tuen Mun Hospital, Hong Kong, China

**Keywords:** damage, lupus, mortality, time trend, morbidity

## Abstract

**Objectives:** To revisit the trend of survival of systemic lupus erythematosus in a cohort of Chinese patients over 25 years.

**Methods:** Patients who fulfilled the 1997 ACR criteria for SLE and were followed in our hospital since 1995 were included. Patients were stratified into two groups according to the year of diagnosis: (1) 1995–2004 and (2) 2005–2018. Survival of patients was studied by Kaplan–Meier analysis. Organ damage as assessed by the Systemic Lupus International Collaborating Clinics (SLICC) damage index (SDI) and causes of death in the first 10 years of SLE onset was compared between the two groups. Cox regression was used to study factors associated with survival.

**Results:** A total of 1,098 SLE patients were registered in our database. After excluding 157 patients diagnosed outside the time period of 1995–2018, 941 patients were studied (92% women). All were ethnic Chinese. The mean age of SLE onset was 35.1 ± 14.4 years, and the mean duration of observation was 13.1 ± 6.6 years. Seventy-seven (8.2%) patients were lost to follow-up. Groups 1 and 2 consisted of 364 and 577 patients, respectively. The mean SDI score at 10 years of disease onset was significantly higher in group 1 than group 2 patients (1.01 ± 1.43 vs. 0.57 ± 0.94; *p* < 0.01), particularly in the neuropsychiatric, musculoskeletal, and gonadal domains. Within 10 years of SLE onset, 32 (8.8%) patients in group 1 and 25 (4.3%) patients in group 2 died (*p* = 0.005). The 5- and 10-year cumulative survival rates were 93.6 and 91.0% in group 1 and 96.5 and 94.2% in group 2 patients, respectively (log-rank test *p* = 0.048). Infection accounted for more than half of the deaths in both groups. More group 1 than group 2 patients died of vascular events, but the difference was not statistically significant. Cox regression showed that the age of SLE onset and damage score accrued at 10 years, but not the time period in which SLE was diagnosed, were significantly associated with mortality.

**Conclusions:** The improvement in survival of our SLE patients is probably related to the accrual of less organ damage in the past 15 years.

## Introduction

Systemic lupus erythematosus (SLE) is a multisystem autoimmune disease that predominantly affects younger women. The disease course is characterized by periods of remission and exacerbation that are largely unpredictable (1). The consequence of this fluctuating disease course is organ damage, as a result of persistent disease activity or treatment-related complications. Organ damage in SLE is a major risk factor for further organ damage accrual, impaired quality of life (QOL), and mortality (2, 3).

With earlier referral and diagnosis, availability of renal replacement therapy, more potent antimicrobial drugs, less toxic immunosuppressive regimens, and improved supportive care for organ complications, the survival of SLE has improved tremendously in the past few decades (4). A recent meta-analysis (5) of 125 studies showed that survival of adult SLE improved gradually from the 1950s to the mid-1990s in both affluent and less affluent countries. However, the survival rate has plateaued since the mid-1990s despite a reduced proportion of patients deceased due to active SLE. A substantial proportion of SLE patients still succumbed of complications related to refractory disease or therapies (6). The mortality of SLE is increased by at least three- to 4-fold when compared with the age- and gender-matched population (7). The commonest cause of death is infection, followed by vascular complications and cancer. Thus, there are unmet needs to introduce more effective but less toxic therapies in SLE and reduce long-term complications such as atherosclerosis, osteoporosis, and malignancies in order to improve the survival of the disease further.

Although there have been a number of survival studies of SLE in the past decade, not too many were performed in the Asian populations (8–25). Analysis of the survival of hospitalized patients (9, 22), those admitted to the intensive care unit (12, 19) or referred to tertiary centers (23), subsets of patients with nephritis (14), neuropsychiatric manifestations (17), or pulmonary hypertension (15) in some studies would create bias in the mortality rate. Moreover, the follow-up of most of these Asian studies was not long enough to look at the trend of survival over time. A large cohort of Chinese SLE patients in our hospital was followed by the same group of physicians since 1995. We hereby report the secular trend of survival of these patients in the past 25 years.

## Patients and Methods

Tuen Mun Hospital is a large regional public hospital in Hong Kong providing medical services to a population of 1.2 million residing in the vicinity. All citizens are entitled to receive a full range of medical services from government hospitals by payment of a nominal fee. Patients diagnosed to have SLE after 1990 in our outpatient clinics or during hospital stay or referred from other hospitals are captured in a longitudinal database. Adult patients under the care of all specialists such as rheumatologists, nephrologists, geriatricians, hematologists are included. All are ethnic Chinese with their family origin in southern part of China. All patients fulfill four or more 1997 American College of Rheumatology (ACR) criteria for the classification of SLE (26) and are being followed by the same group of physicians at an usual interval of 12–16 weeks. More frequent clinic visits are arranged for those with active/unstable disease or complications.

The demographic characteristics, cumulative manifestations of SLE, and autoantibodies of the patients are captured. The clinical status of the SLE patients in our registry is updated every 6 months.

### Assessment of Organ Damage and Mortality

Organ damage of SLE is assessed by the Systemic Lupus International Collaborating Clinics (SLICC) damage index (SDI) (27), a validated instrument consisting of 41 items that measure irreversible organ damage not caused by active inflammation in 12 organ systems. Each item should be present for at least 6 consecutive months in order to be scored. The SDI score is updated annually.

For patients who died during their disease course, we analyzed the causes of death according to the documentation of their attending specialists in the medical records based on investigation results or best clinical judgment. Autopsy would be performed for uncertain cause of death, academic interest, or medico-legal purpose. For those who succumbed due to any causes, data were censored at the time of death.

### Trend of Survival Over Time

To study the trend of survival over time and the causes of death, we divided our patients arbitrarily into two groups: (1) group 1: SLE diagnosed between 1995 and 2004; and (2) group 2: SLE diagnosed between 2005 and 2018. Organ damage, mortality, and causes of death in the first 10 years of SLE diagnosis were also compared between the two groups.

### Statistical Analyses

Unless otherwise stated, values in this study were expressed as mean ± SD (standard deviation). Comparison of continuous variables between two groups was performed using the independent sample Students' *t*-test. Categorical variables were compared by the chi-square test. When the frequency was <5 in any cell of the contingency table, the Fisher exact test was used. Correction for multiple comparisons was made by Bonferroni's method. The cumulative probability of survival of the patients over time was studied by Kaplan–Meier analysis. For those who died or were lost to follow-up, data were censored at the time of death and last clinic visits or hospitalization, respectively. Comparison of survival between two groups was made by the long-rank test. Cox regression was used to analyze the hazard ratio (HR) and 95% confidence interval (CI) for survival. Covariates included in the model were age of SLE onset, sex, SDI score, renal involvement, ever use of hydroxychloroquine (HCQ) within 10 years of diagnosis, and time period in which patients were diagnosed. Statistical significance was defined as a *p*-value of <0.05, 2-tailed. All statistical analyses were performed using the SPSS program (version 18.0 for Windows 10).

## Results

### Study Population and Clinical Manifestations

Up to March 2020, a total of 1,098 SLE patients were registered in our cohort database. One hundred and fifty-seven patients were excluded as the diagnosis of SLE was made before 1995 or after 2019. Finally, 941 patients were included (862 women; 92%). All were ethnic Chinese. The mean age of onset of SLE was 35.1 ± 14.4 years, and the mean follow-up of these patients was 13.1 ± 6.6 years. Seventy-seven (8.2%) patients were lost to follow-up, and their data were censored at the last clinic visits.

There were 364 patients in group 1 and 577 patients in group 2. The cumulative manifestations of these patients are shown in Table 1. The age of onset of SLE was significantly higher in group 2 patients. The prevalence of anti-ENA antibodies was also significantly higher in this group. Regarding clinical manifestations, arthritis and lymphopenia were significantly more frequent in group 1 patients. The frequencies of other manifestations were similar between the two groups. Regarding treatment of SLE during the first 10 years of diagnosis, more group 2 patients had received HCQ and mycophenolate mofetil (MMF) whereas more group 1 patients were ever treated with cyclophosphamide (CYC).

**Table 1:** Cumulative manifestations and therapies within 10 years of diagnosis.

**Clinical manifestations**	**Group 1 (*N* = 364)**	**Group 2 (*N* = 577)**	***P***	^**^P
	***N*** **(%); mean ± SD**			
Age of onset, years	32.4 ± 13.6	36.8 ± 14.6	<0.001	<0.03
Women	328 (90.1)	534 (92.5)	0.19	NS
Duration of follow-up, years	20.2 ± 2.7	8.6 ± 3.9	<0.001	<0.03
Arthritis	263 (72.2)	339 (58.8)	<0.001	<0.03
Malar rash	173 (47.5)	243 (42.1)	0.10	NS
Discoid rash	44 (12.1)	50 (8.7)	0.09	NS
Mucosal ulceration	58 (15.9)	78 (13.5)	0.31	NS
Photosensitivity	100 (27.5)	110 (19.1)	0.003	NS
Hemolytic anemia	92 (25.3)	135 (23.4)	0.51	NS
Leukopenia	147 (40.4)	185 (32.1)	0.009	NS
Thrombocytopenia	92 (25.3)	132 (22.9)	0.40	NS
Lymphopenia	264 (72.5)	344 (59.6)	<0.001	<0.03
Lymphadenopathy	60 (16.5)	85 (14.7)	0.47	NS
^*^Neuropsychiatric manifestations	43 (11.8)	50 (8.7)	0.12	NS
Renal	211 (58.0)	282 (48.9)	0.007	NS
Serositis	73 (20.1)	117 (20.3)	0.93	NS
Myositis	13 (3.6)	21 (3.6)	0.96	NS
Gastrointestinal	25 (6.9)	60 (10.4)	0.07	NS
**Autoantibodies**				
Anti-dsDNA	252 (69.2)	400 (69.3)	0.98	NS
Anti-Sm	46 (12.6)	160 (27.7)	<0.001	<0.03
Anti-Ro	205 (56.3)	384 (66.6)	0.002	0.06
Anti-La	50 (13.7)	152 (26.3)	<0.001	<0.03
Anti-nRNP	95 (26.1)	220 (38.1)	<0.001	<0.03
**Medications ever used ≥1 month**				
Prednisolone	280 (76.9)	468 (81.1)	0.12	NS
Hydroxychloroquine	213 (58.5)	452 (78.3)	<0.001	<0.03
Methotrexate	23 (6.3)	64 (11.1)	0.01	NS
Mycophenolate mofetil	66 (18.1)	227 (39.3)	<0.001	<0.03
Tacrolimus/	78 (21.4)	136 (23.6)	0.45	NS
Cyclophosphamide	87 (23.9)	47 (8.1)	<0.001	<0.03
Azathioprine	200 (54.9)	276 (47.8)	0.03	NS

*N, number; SD, standard deviation; NS, non-significant*.

* *Only included manifestations that required immunosuppression (e.g., psychosis, acute confusional state, myelitis, neuropathy, myasthenia gravis)*.

** *P-values adjusted by Bonferroni's method*.

### Organ Damage

Table 2 shows the organ damage scores in the two groups of patients within 10 years of disease onset. The mean total SDI score was significantly higher in group 1 than group 2 patients. Among the 12 organ systems, the SDI scores in the neuropsychiatric, musculoskeletal, and gonadal domains were significantly higher in group 1. The proportion of patients with organ damage in these three systems was also significantly higher in this group of patients. In those patients with organ damage, the mean time to first damage was 41.1 ± 40 months in group 1 and 18.9 ± 28.1 months in group 2 (*p* < 0.001), suggesting that late damage was more common in group 1 patients.

**Table 2:** Organ damage within 10 years of SLE onset in patients studied.

**Organ/system**	**Group 1**	**Group 2**	^*^P
	***N*** **(%)**		
Ophthalmological	19 (5.2)	24 (4.2)	NS
Neuropsychiatric	54 (14.8)	50 (8.7)	0.042
Renal	39 (10.7)	37 (6.4)	NS
Pulmonary	18 (4.9)	34 (5.9)	NS
Cardiovascular	16 (4.4)	23 (4.0)	NS
Peripheral vascular	10 (2.7)	13 (2.3)	NS
Gastrointestinal	3 (0.8)	2 (0.3)	NS
Musculoskeletal	50 (13.7)	40 (6.9)	0.014
Dermatological	22 (6.0)	31 (5.4)	NS
Gonadal	17 (4.7)	2 (0.3)	<0.014
Endocrinological	9 (2.5)	15 (2.6)	NS
Malignancy	8 (2.2)	14 (2.4)	NS
Total SDI	167 (45.9)	206 (35.7)	0.028
SDI ≥ 5	10 (2.7)	3 (0.5)	NS
Mean time to first organ damage, months	41.1 ± 40	18.9 ± 28.1	<0.001

*SD, standard deviation; N, number; SDI, systemic lupus; NS, non-significant; Erythematosus international collaborating clinics (SLICC) damage index*.

* *P-values adjusted by Bonferroni's method*.

### Mortality

Within 10 years of SLE onset, 32 (8.8%) patients in group 1 and 25 (4.3%) patients in group 2 died (*p* = 0.005). Table 3 shows the causes of death in these patients. Infection was the main cause of death in both groups, accounting for more than half of the deaths. Vascular causes of death were more common in group 1 than group 2 patients, but the difference was not statistically significant.

**Table 3:** Causes of death within 10 years of onset of SLE.

**Cause of death**	**Group 1**	**Group 2**	***P***
	**Number (%)**		
Infection	18 (56.3)	14 (56.0)	0.99
^*^Vascular	5 (15.6)	1 (4.0)	0.22
Pulmonary hypertension	1 (3.1)	3 (12.0)	0.31
Malignancy	2 (6.3)	3 (12.0)	0.65
Refractory and uncontrolled SLE	2 (6.3)	1 (4.0)	1.00
Suicide	1 (3.1)	1 (4.0)	1.00
Sudden death without obvious causes	3 (9.4)	2 (8.0)	1.00
Total	32 (100)	25 (100)	0.005

* *Included cerebrovascular accident, acute coronary syndrome, and aortic dissection*.

**Figure 1:**
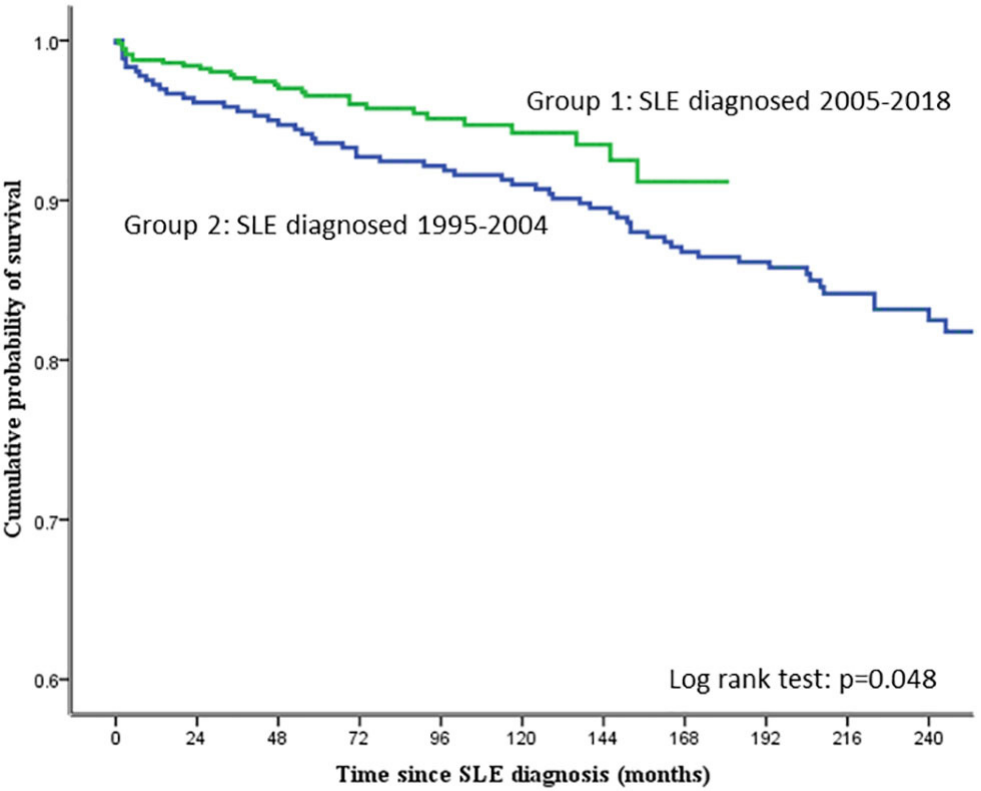
Cumulative probability of survival in the SLE patients studied.

The cumulative survival rate of all the 941 patients studied was 95.3, 92.9, 88.5, and 84.5% at 5, 10, 15, and 20 years, respectively. Figure 1 shows the cumulative probability of survival of the two groups of patients. The 3-, 5-, and 10-year survival in group 1 patients was 95.6, 93.6, and 91%, respectively. The corresponding figures for group 2 were 97.7, 96.5, and 94.2%, respectively. The difference in survival between group 1 and group 2 was statistically significant (log-rank test; *p* = 0.048).

Table 4 shows the causes of death of all the 941 patients studied according to the duration of SLE. In addition to the 57 patients who died within 10 years of SLE onset, 30 other patients in the cohort died beyond 10 years of SLE onset (total 87 deaths). Patients who died beyond 10 years of disease onset were less likely to be caused by infection but more likely to be contributed by pulmonary hypertension, chronic cardiopulmonary disease, and malignancies.

**Table 4:** Cause of death of the whole cohort of SLE patients.

**Causes of death**	**0–5 years of onset**	**>5–10 years of onset**	**>10 years of onset**
**Number of deaths**	***N*** **= 40**	***N*** **= 17**	***N*** **= 30**
Infection	24 (60%)	8 (47%)	6 (20%)
Vascular	4 (10%)	2 (11.8%)	6 (20%)
Pulmonary hypertension	2 (5%)	2 (11.8%)	5 (16.7%)
Malignancy	3 (7.5%)	2 (11.8%)	5 (16.7%)
Sudden death without obvious causes	2 (5%)	3 (17.6%)	5 (16.7%)
Suicide	2 (5%)	0 (0%)	0 (0%)
Refractory/uncontrolled SLE	3 (7.5%)	0 (0%)	1 (3.3%)
Pulmonary fibrosis	0 (0%)	0 (0%)	2 (6.7%)

*SLE, systemic lupus erythematosus*.

Table 5 shows the Cox regression analysis for factors associated with mortality. Univariate analysis showed that age of onset, male sex, renal involvement, and SDI score at 10 years were associated with mortality. The use of HCQ was negatively associated with mortality. Group 1 patients showed a higher mortality rate than group 2, but statistical significance was borderline (hazard ratio 0.61 [0.38–1.001]; *p* = 0.05). Multivariate analysis revealed only the age of onset (1.06 [1.04–1.08] per year; *p* < 0.001), SDI at 10 years (1.65 [1.47–1.85] per point; *p* < 0.001), and ever use of HCQ (0.54 [0.34–0.85]; *p* = 0.008) were significantly associated with mortality. The time period in which SLE was diagnosed was not a significant factor determining this outcome.

**Table 5:** Cox regression analysis of factors affecting mortality.

**Covariates**	**Univariate**	***P***	**Multivariate**	***P***
	**Hazard ratio (95% confidence interval)**		**Hazard ratio (95% confidence interval)**	
Age of SLE onset, per year	1.06 (1.05–1.08)	<0.001	1.06 (1.04–1.08)	<0.001
SDI score at 10 years, per point	1.45 (1.32–1.59)	<0.001	1.60 (1.42–1.80)	<0.001
Male sex	2.29 (1.31–4.00)	0.004	1.30 (0.73–2.31)	0.38
Renal involvement	2.04 (1.37–3.02)	<0.001	1.36 (0.86–2.17)	0.19
Ever use of HCQ at 10 years	0.42 (0.28–0.63)	<0.001	0.54 (0.34–0.85)	0.008
Group 2 (vs. group 1)	0.61 (0.38–1.001)	0.05	0.76 (0.45–1.28)	0.30

*SDI, systemic lupus erythematosus international collaborating clinics (SLICC) damage index; HCQ, hydroxychloroquine*.

## Discussion

This is a survival study of an inception cohort of SLE patients diagnosed since 1995. Our results showed that the overall 10-year survival of our SLE patients was 92.9%, which is similar to those reported in Asian studies after the 2000s (9–11, 16, 18, 21, 24). However, direct comparison is not feasible because the cumulative survival rate computed by the Kaplan–Meier method was not reported in most of these studies. In our cohort, there was an improvement in survival of patients diagnosed between 2005 and 2018 compared to those diagnosed between 1995 and 2004. Organ damage was also significantly less common in patients diagnosed in the more recent period. Univariate Cox regression analysis showed that survival of the group of patients diagnosed after 2004 (group 2) was better than those who diagnosed earlier (group 1) (*p* = 0.05; borderline significance). However, statistical significance was lost when damage score at 10 years, sex, age of SLE onset, renal involvement, and ever use of HCQ were put into the multivariate model. As group 1 patients had younger onset of SLE (favorable factor for survival), the worse prognosis of this group was likely due to more organ damage accrued at 10 years when compared to group 2 patients.

An interesting observation was noted when we compared the clinical manifestations of the two groups of patients. Patients diagnosed after 2004 were significantly older at the time of SLE diagnosis, and they were less likely to have arthritis and renal disease during the course of their illness. The mean age of SLE diagnosis has increased from 32.4 years to 36.8 years in the period of 2005–2018. This is consistent with our clinical impression that more SLE patients in the recent decade were diagnosed in the middle age range with the presentation of hematological in the absence of musculoskeletal or dermatological symptoms. The reason for this observation is unclear, but it is unlikely to be the effect of increased referrals or awareness of SLE by primary care physicians as our hospital has been the only specialty referral hospital for SLE in the areas covered by public medical service in the past 2–3 decades. Moreover, there has not been any change in the pattern of inter-specialty referral and our cohort of SLE patients has included all the patients seen by different specialists in our department. One postulation is the increased use of HCQ in our SLE patients beyond dermatological and articular indications in the past decade, which might protect against the development of joint and renal manifestations. However, further clinical trials of HCQ are needed to confirm this postulation.

As shown in Table 1, the prevalence of antibodies to the anti-extractable nuclear antigens (anti-ENA) has increased from the period 1995–2004 to 2005–2018. This can be explained by the change in the methodology of anti-ENA assay in our laboratory from counter immune-electrophoresis (CIEP) to enzyme-linked immunosorbent assay (ELISA) followed by confirmation with Western blotting in the year 2005.

Another observation from the current study is that patients diagnosed after 2004 had accrued less organ damage at 10 years, which is the most important factor for the improved survival. In particular, reduced damage in the neuropsychiatric, musculoskeletal, and gonadal domains has made the difference. In a cohort study from the US John Hopkins University, glucocorticoid use was a major risk factor for organ damage accrual (28). The risk of organ damage was increased by more than 3-fold when the mean daily dosage of prednisone was >20 mg. The multicenter Asia Pacific lupus collaboration group also reported that the time-adjusted mean prednisolone dose was independently associated with damage accrual in SLE patients (29). In a subset of patients with no disease activity over time, the mean prednisolone dose remained an independent risk factor for damage accrual. Thus, every attempt should be made to limit the dosage and duration of glucocorticoid use in SLE. The more judicious use of high-dose glucocorticoids in our cohort has led to the observed reduction in avascular necrosis of the bone and osteoporotic fractures. For instance, the standard dosage of prednisolone for severe lupus nephritis has reduced from 1 mg/kg/day to around 0.6–0.8 mg/kg/day in recent years with the early use of glucocorticoid-sparing agents such as azathioprine and mycophenolate mofetil (MMF). Moreover, the duration of high-dose prednisolone has been limited to <8 weeks. On the other hand, the substitution of cyclophosphamide (CYC) pulses to MMF as first-line induction therapy of major organ disease in most patients is linked to the lower incidence of premature ovarian failure in our patients. With the increased awareness of the cardiovascular complications, regular surveillance of traditional risk factors is being performed in recent years, which serves to explain the lower incidence of vascular complications such as ischemic stroke in our patients.

As shown in our data, infection remains the main cause of death of our SLE patients regardless of the time period of diagnosis. Thus, more effort has to be done to minimize the risk of infection in SLE patients. Apart from the use of the minimally effective doses of immunosuppressive medications, measures to prevent common viral and bacterial infections are equally important. Influenza and pneumococcal vaccines, which are safe and efficacious in SLE (30), are not routinely administered to our patients. These vaccinations should be more encouraged in the future by a standard protocol facilitated by our rheumatology nurses. Personal hygiene and social distancing are crucial as reflected in the COVID-19 global epidemic. Antibiotic prophylaxis for certain opportunistic infections such as pneumocystis jirovecii pneumonia (31) is not yet a routine practice in Asian countries but should be considered for SLE patients with multiple risk factors such as renal dysfunction, severe lymphopenia, and treatment with combination of multiple immunosuppressive agents, particularly high-dose glucocorticoid and CYC.

There are several limitations in this study. First, we did not have data on the serial disease activity score over time and therefore it is uncertain if better disease control in recent years has contributed to the improved survival. Second, data on the cumulative dosages of medications, particularly prednisolone and HCQ, are unavailable for evaluation of their contribution to SLE prognosis. Finally, we did not have information of all the patients on the duration of symptoms before SLE diagnosis and their drug compliance, which might also be factors affecting the long-term prognosis.

## Conclusion

We have presented the data of a large cohort of SLE patients treated in a service hospital over 25 years and confirmed the improved survival. The improvement in SLE prognosis is mainly contributed by a significant reduction in organ damage accrual, which is linked to the more judicious use of glucocorticoids, early use of glucocorticoid-sparing agents, and the regular surveillance and treatment of traditional vascular risk factors. Despite the decrease in the mortality figures, further reduction should be the target. As infection remains the main cause of death, protocol-based vaccination programs against common viral and bacterial infections, as well as antibiotic prophylaxis for opportunistic infections in high-risk patients, should be adopted in the future. It is hoped that SLE patients can continue to live longer with less end organ damage accrual so that they can enjoy a better quality of life.

## Data Availability Statement

The raw data supporting the conclusions of this article will be made available by the authors, without undue reservation.

## Ethics Statement

The studies involving human participants were reviewed and approved by Ethics committee, Tuen Mun Hospital, Hong Kong. Written informed consent for participation was not required for this study in accordance with the national legislation and the institutional requirements.

## Author Contributions

All authors have contributed to the interpretation of the data and approved the final manuscript.

## Conflict of Interest

The authors declare that the research was conducted in the absence of any commercial or financial relationships that could be construed as a potential conflict of interest.
